# Supplementary material for: Accuracy of Tumour-Associated Circulating Endothelial Cells as a Screening Biomarker for Clinically Significant Prostate Cancer
Source: Cancers (Basel). 2019 Jul 27;11(8):1064. doi: 10.3390/cancers11081064 (PMC6721410; doi:10.3390/cancers11081064)
Supplement: Supplementary file 1 [file cancers-11-01064-s001.pdf]

Supplementary Materials

Accuracy of Tumour-Associated Circulating Endothelial Cells as a Screening Biomarker for Clinically Significant Prostate Cancer

Sebastian Chakrit Bhakdi, Prapat Suriyaphol, Ponpan Thaicharoen, Sebastian Tobias Karl Grote, Chulaluk Komoltri, Bansithi Chaiyaprasithi and Komgrid Charnkaew

Part A

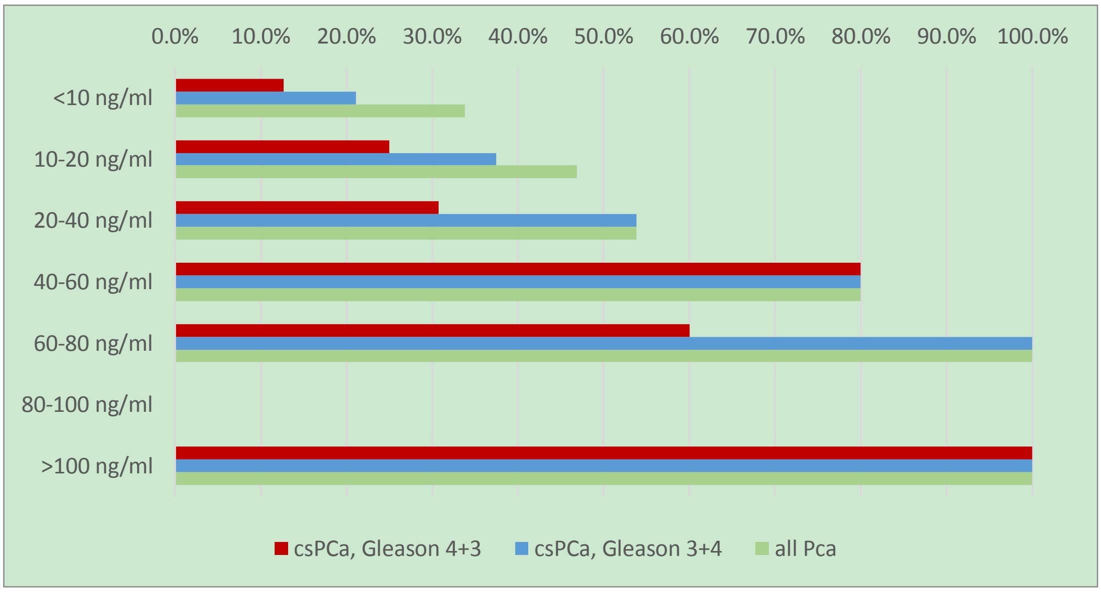

Figure S1. Prevalence of all cancer and clinically significant cancer according to primary and secondary definition (Gleason 4 + 3 and Gleason 3 + 4) in included group of men. Overall results from first and second TRUS biopsies.

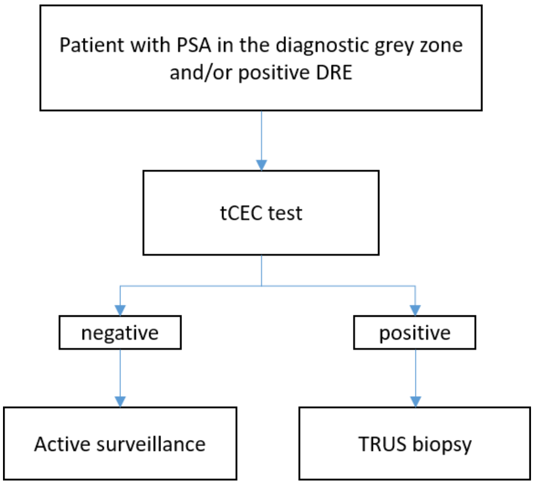

Figure S2. Diagnostic workflow for estimation of clinical utility of using the tCEC test as add-on triage test for patients with elevated PSA and/or positive digital rectal examination.

Table S1. Accuracy of the tCEC test for all PSA ranges.

PSA ng/mL	CEC+ Biopsy+	CEC+ Biopsy-	CEC- Biopsy+	CEC- Biopsy-	Sensitivity	Specificity	PPV	NPV
**All PCa**								
<10	15	13	7	34	68%	72%	54%	83%
10–20	9	6	7	12	56%	67%	60%	63%
20–40	3	3	4	3	43%	50%	50%	43%
40–60	2	0	2	1	50%	100%	100%	33%
60–80	5	0	0	0	100%	NA	100%	NA
80–100	0	0	0	0	NA	NA	NA	NA
>100	15	0	5	0	75	NA	100	0
**total**	**49**	**22**	**25**	**50**				
**csPCa, Gleason ≥ 3 + 4**								
**PSA ng/mL**	**CEC+ Biopsy+**	**CEC+ Biopsy- or csPCa-**	**CEC- Biopsy+**	**CEC- Biopsy- or csPCa-**	**Sensitivity**	**Specificity**	**PPV**	**NPV**
<10	9	19	3	38	75%	67%	32%	93%
10–20	7	8	5	14	58%	64%	47%	74%
20–40	3	3	4	3	43%	50%	50%	43%
40–60	2	0	2	1	50%	100%	100%	33%
60–80	5	0	0	0	100%	NA	100%	NA
80–100	0	0	0	0	NA	NA	NA	NA
>100	15	0	5	0	75%	NA	100%	0%
**Total**	**41**	**30**	**19**	**56**				
**csPCa, Gleason ≥ 4 + 3**								
**PSA ng/mL**	**CEC+ Biopsy+**	**CEC+ biopsy- or csPCa-**	**CEC- Biopsy+**	**CEC- Biopsy- or csPCa-**	**Sensitivity**	**Specificity**	**PPV**	**NPV**
<10	5	23	2	39	71%	63%	18%	95%
10–20	4	11	4	15	50%	58%	27%	79%
20–40	1	5	3	4	25%	44%	17%	57%
40–60	2	0	2	1	50%	100%	100%	33%
60–80	3	2	0	0	100%	0%	60%	NA
80–100	0	0	0	0	NA	NA	NA	NA
>100	15	0	5	0	75%	NA	100%	0%
**Total**	**30**	**41**	**16**	**59**				

Table S2. Number of patients who received a second TRUS biopsy during active surveillance and tCEC test results.

PSA Reading (ng/mL)	Negative 1st Biopsy	Received 2nd Biopsy	of Which Originally CEC+	CEC+, 2nd Biopsy+, All Pca	CEC-, 2nd Biopsy+, All Pca	CEC+, 2nd Biopsy+, Gleason ≥ 4 + 3	CEC+, 2nd Biopsy+, Gleason ≥ 3 + 4
**<10**	49	8	5	2	0	2	2
**10–20**	19	4	3	1	0	1	1
**20–40**	7	4	3	1	0	0	1
**40–60**	1	0	0	0	0	0	0
**60–80**	0	0	0	0	0	0	0
**80–100**	0	0	0	0	0	0	0
**>100**	1	1	1	1	0	1	1

Table S3. Potential implications of adding the tCEC test to the screening workflow for prostate cancer.

A) Relative Reduction of Over-Diagnosis According to the Primary Definition

PSA ng/mL	Screening with PSA Alone		Screening with tCEC Triage Test		
	Primary biopsies	over-diagnosed Gleason 3 + 4	over-diagnosed Gleason 3 + 4	relative reduction of overdiagnosis	CI 95%
<10	69	10	6	40%	
10–20	34	4	2	50%	
20–40	13	0	0	0%	
40–60	5	0	0	0%	
60–80	5	0	0	0%	
80–100	0	0	0	NA	
>100	20	0	0	0%	

B) Relative Reduction of Over-Diagnosis According to the Secondary Definition

PSA ng/mL	Screening with PSA Alone		Screening with tCEC Triage Test		
	Primary biopsies	over-diagnosed Gleason 4 + 3	over-diagnosed Gleason 4 + 3	relative reduction of overdiagnosis	CI 95%
<10	69	15	10	33%	
10–20	34	8	5	38%	
20–40	13	3	2	33%	
40–60	5	0	0	0%	
60–80	5	2	2	0%	
80–100	0	0	0	NA	
>100	20	0	0	NA	

C) Reduction of Total Biopsies

PSA ng/mL	Screening with PSA Alone	Screening with tCEC Triage Test		
	Primary biopsies	biopsies saved	reduction of total biopsies	CI 95%
<10	69	41	59%	
10–20	34	19	56%	
20–40	13	7	54%	
40–60	5	3	60%	
60–80	5	0	0%	
80–100	0	0	NA	
>100	20	5	25%	

Part B

Material and Reagents

1. Red and White Blood Cell Depletion of Heparinised Whole Blood Samples

Equipment and Materials Required:

hMX™ Lysis buffer	X-Zell, Singapore
PBS/EDTA 5mM/ FBS 1%	Biochrom, Berlin, Germany
hMX™ anti-Biotin beads	X-Zell, Singapore
hMX™ Columns 1.5g	X-Zell, Singapore
hMX™ flow resistor	X-Zell, Singapore
hMX™ Priming solution	X-Zell, Singapore
hMX™ Separation buffer	X-Zell, Singapore
Washing buffer	X-Zell, Singapore
FC Block	Biolegend, San Diego, CA, USA
D-Biotin 0.05%	X-Zell, Singapore
Blocking Buffer I	X-Zell, Singapore
Anti-CD45 biotin	Exbio, Prague, Czech Republic
Anti-CD235a biotin	Ebioscience, San Diego, CA, USA
Purified anti-biotin antibody	Biolegend, San Diego, CA, USA

2. Cryoimmunostaining

Equipment and Materials Required:

Cytofuge 2	Statspin, Atlanta, GA, USA
Cryofixation Station	X-Zell, Singapore
Cryostainer	X-Zell, Singapore
Cytocentrifuge Buffer	X-Zell, Singapore
Cryostaining buffer	X-Zell, Singapore
Blocking buffer I	X-Zell, Singapore
Blocking buffer II	X-Zell, Singapore
FC block	Biolegend, San Diego, CA, USA
CapGap clips	X-Zell, Singapore
Cell-adhesive slides	X-Zell, Singapore
Cryofixation Buffer I	X-Zell, Singapore
Cryofixation Buffer II	X-Zell, Singapore
Slide Fixation cartridges	X-Zell, Singapore
Priming solution II	X-Zell, Singapore
Premount buffer	X-Zell, Singapore
Mounting Buffer	X-Zell, Singapore

**Fluorophore**	**Antibody**	**Clone**	**Source**	**Isotype**
BV421	CD34	581	BD	Mouse IgG1
PO	CD45	HI30	EXB	Mouse IgG1
AF488	Vimentin	EPR3776	ABC	Rabbit IgG
PE	Pan-Cytokeratin	C-11	ABC	Mouse IgG1
PE	CD326 (EpCAM)	VU-1D9	EXB	Mouse IgG1
AF594	CD31	WM59	BLG	Mouse IgG1
DRAQ5	Nuclear Dye	-	BST	-

Abbreviations: AF = AlexaFluor, BV =BrilliantViolet, PB = Pacific Blue, PO =Pacific Orange. ABC = Abcam, Cambridge, UK; BD = BD Bioscience, San Jose, USA; BLG = Biolegend, San Diego, CA, USA; EXB = exbio, Czech Republic; BST = Biostatus, Leicestershire, UK.
